# Acupuncture for the Treatment of Liver Cirrhosis: A Meta-analysis

**DOI:** 10.1155/2020/4054781

**Published:** 2020-11-27

**Authors:** Lu Qi, Shuang Li, Jun Xu, Jie Xu, Wangzouyang Lou, Liangbin Cheng, Chizhi Zhang

**Affiliations:** ^1^Hubei University of Chinese Medicine, Wuhan 430061, China; ^2^The Second People's Hospital of Yichang, Yichang 443000, China; ^3^Hubei Provincial Hospital of TCM, Wuhan 430061, China

## Abstract

Acupuncture is widely used in the clinical treatment of liver cirrhosis (LC) in China. However, the efficacy of acupuncture on LC has not been fully confirmed by systematic analysis. This current meta-analysis evaluated the impact effect of acupuncture on patients with LC. We conducted a systematic literature search of the China National Knowledge Infrastructure, the Chinese Biomedical Database (SinoMed), VIP medicine information system, Wanfang Data, PubMed, Cochrane Library, Web of Science, and Embase. Further, we used Review Manager 5.3 software for the analysis of the data and Stata 14.0 software for the Egger test to assess publication bias. Fifteen studies involving 1066 patients were included in the meta-analysis. The primary outcome was the efficacy rate of acupuncture therapy. The secondary outcomes were impact on acupuncture on liver function grading assessment and lab tests related to liver functions. The result suggested that acupuncture is an effective treatment option for patients with LC as a complementary therapy. However, the recommendation is weak due to some limitations of the included studies.

## 1. Introduction

Liver cirrhosis (LC), which is characterized by the formation of diffuse fibrous pseudolobules and the proliferation of blood vessels inside and outside the liver, is an advanced liver disease caused by various chronic liver diseases. LC is a cause of rising mortality and morbidity. Globally, 1.16 million people die from LC every year [1]. According to epidemiological surveys, hepatitis B virus (HBV) is the leading cause of LC in most parts of Asia and sub-Saharan Africa; alcohol abuse, hepatitis C virus (HCV), and nonalcoholic liver diseases are the main causes in developed countries [2]. At present, the primary treatment strategy includes cause-specific interventions and prevention of encephalopathy, portal hypertension, variceal bleeding, ascites, and other complications. The main treatment goals are to stop disease progression, improve the quality of life, and prolong survival time. Although numerous studies are being conducted, no drug on reversal of the disease is yet approved by the Food and Drug Administration for treating LC [3]. A lack of treatments for LC patients with decompensation or poor liver function makes LC a life-threatening disease and a major cause of death worldwide.

In recent years, studies and clinical observations have shown that traditional Chinese medicine is effective in the treatment of liver diseases, especially in the field of LC. As an important complementary and alternative medicine, acupuncture has a history of thousands of years in China. It has been proved to be effective and widely used in clinical treatment of hepatic diseases, such as nonalcoholic fatty liver disease (NAFLD) [4], chronic hepatitis B (CHB) [5], and LC [6]. Both ancient literature and modern scientific evidence showed that acupuncture, as a kind of complementary and alternative therapy, has a positive effect on LC. For many years, it is one of the therapy methods of LC in China. In addition, the effect of acupuncture on LC has been documented in numerous animal and clinical studies. Multiple mechanisms have been suggested to contribute to the therapeutic effect of acupuncture, including anti-inflammation effects and immunomodulatory and neurotransmitter regulation [7, 8]. Reviewing current studies of acupuncture on LC treatment may provide opportunities to develop better therapeutic strategies for it. In view of the wide usage of acupuncture, we conducted a meta-analysis to summarize the effect of acupuncture on LC.

## 2. Materials and Methods

### 2.1. Search Strategy

The meta-analysis was carried out according to the Preferred Reporting Items for Systematic Reviews and Meta-Analyses (PRISMA) guidelines [9]. Randomized controlled trials (RCTs) on the efficacy of acupuncture against LC were searched by two authors independently until June 2020. The Chinese database mainly included the Chinese Biomedical Database (SinoMed or CMB), the China National Knowledge Infrastructure (CNKI), the VIP medicine information system (VIP), and Wanfang Data (WANFANG). English databases included PubMed, Embase, the Cochrane Library, and Web of Science. We also searched the Chinese Clinical Trial Registry (http://http://www.chictr.org.cn/), ClinicalTrials.gov (https://www.clinicaltrials.gov/), and the World Health Organization International Clinical Trials Registry Platform (https://www.who.int/ictrp/en/) for unpublished trials. The following Medical Subject Headings (MeSH) terms and free text were used: “acupuncture”, “liver cirrhosis”, and “randomized controlled trial”. Detailed search strategy is provided in the supplementary data (available here). We contacted the principal authors for any missing information.

### 2.2. Study Selection

Two reviewers independently screened the literature according to the inclusion criteria and exclusion criteria. We included randomized clinical trials that did not limit publication status and blinding. We allowed cointervention when it was applied equally to the experimental group and the control group. PICOS criteria for study selection are shown in Table 1.

The inclusion criteria were as follows: (a) RCTs with the acupuncture intervention group and the control group; (b) cointerventions which were also allowed when the cointerventions were administered equally to all intervention groups; (c) studies including clear diagnostic criteria and efficacy evaluation criteria; (d) patients with liver cirrhosis, regardless of age or gender; and (e) studies written in English or Chinese.

The exclusion criteria were as follows: (a) republished studies with the same data; (b) studies containing no original data; (c) animal experiments, reviews, case reports, or theoretical literature; (d) if there was no information on diagnostic or efficacy criteria; and (e) if complete data could not be obtained after much effort.

### 2.3. Data Extraction

In order to ensure the integrity and eligibility of the extracted data, two reviewers independently screened the literature back-to-back according to the inclusion and exclusion criteria. In case of disagreement, the literature was evaluated by a third reviewer, and consensus was reached through consultation. The following information included general information, diagnostic criteria, efficacy evaluation criteria, outcome indicators, and adverse reactions. The general information included author, year, and the number of participants and details of intervention. The primary outcome was the efficacy rate of acupuncture therapy. The secondary outcomes were impact of acupuncture on liver function grading assessment and outcomes related to liver function, such as alanine aminotransferase (ALT), aspartate aminotransferase (AST), Albumin (ALB), and total bilirubin (TBIL).

### 2.4. Quality Assessment

The risk of bias was evaluated using the Cochrane system, according to the following six items: random sequence generation, allocation concealment, blinding of participants and personnel, blinding of outcome assessments, incomplete outcome data, selective reporting, and other biases. For each included study, the above six elements were evaluated sequentially with three levels of low, high, and unclear.

### 2.5. Statistical Analysis

A meta-analysis was performed using RevMan5.3 software provided by the Cochrane system. Relative risk (RR) was adopted for dichotomous variables and the mean difference (MD) and 95% confidence interval (CI) for the continuous variables. We tested heterogeneity using the *I* square (*I*^2^) and *P* value (*P*). *P* < 0.05 or *I*^2^ > 50% was considered to indicate substantial heterogeneity, and a random-effects model was used for calculation. Otherwise (*P* ≥ 0.05 or *I*^2^ ≤ 50%), a fixed effects model was used. The Stata 14.0 software was used for the Egger test to assess publication bias.

## 3. Results

### 3.1. Inclusion Study

**Figure 1:**
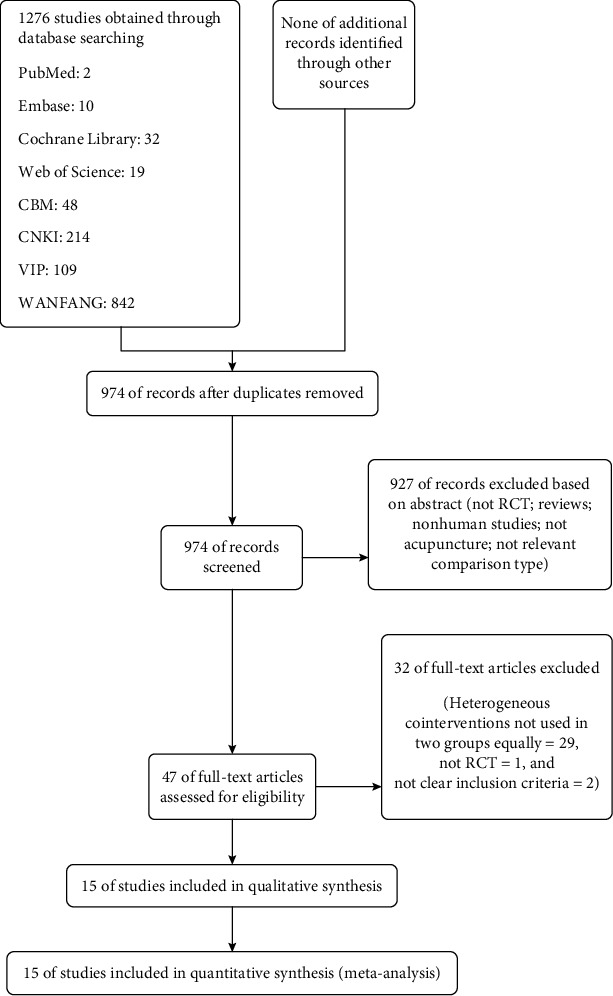
Flowchart of study selection.

The process of literature retrieval and study selection is shown in Figure 1. A preliminary search retrieved 1276 studies. After further screening of these studies, 16 studies were finally included [10–24].

### 3.2. Study Characteristics

The 15 selected studies included a total of 1066 patients and were all conducted in China (Table 2). Their trials compared the effects of acupuncture versus no acupuncture. Heterogeneous cointerventions were used in all trials and were equally used in the control group. In all trials, the control groups received a conventional comprehensive treatment (such as antivirus therapy and liver protection), while the experimental groups received acupuncture therapy combined with the same intervention methods as the control groups. All the studies compared manual needle acupuncture with nonintervention. Two of the studies involved infrared therapeutic apparatus, which were used equally in both groups (chen2017; deng2019). One study involved traditional Chinese medicine decoction, which was administered equally to all intervention groups (xia2019). Three of the fifteen studies received national or provincial or municipal academic funding, while the rest did not report information about funding.

### 3.3. Quality of Study

**Figure 2:**
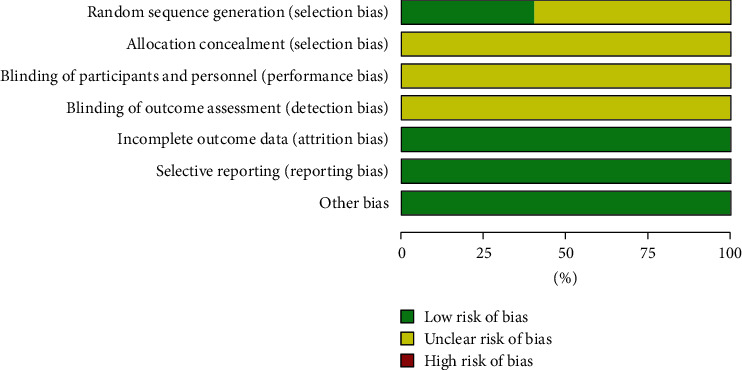
Risk of bias graph: review of authors' judgments regarding each risk of bias item presented as percentages across all included studies.

None of the 15 studies mentioned the use of blinding or allocation concealment. No studies reported participants dropping out or incomplete data. All of these studies were randomized. Four of them mentioned grouping by adopting a random number table and two by drawing lots, and the remainder did not specify the method of sequence generation (Figure 2).

## 4. Meta-Analysis

### 4.1. Efficacy Rate

**Figure 3:**
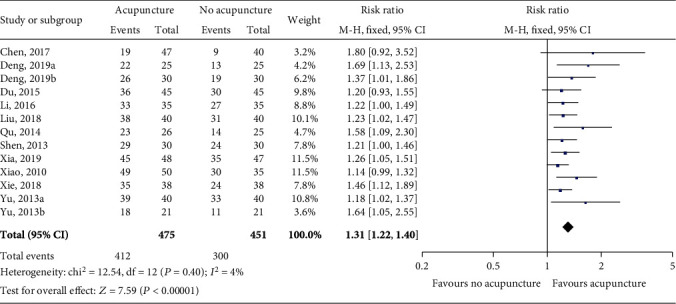
The efficacy rate of acupuncture versus no acupuncture. Both *I*^2^ and *P* are used as the criteria for heterogeneity test. ♦: pooled relative risk; —■—: relative risk and 95% CI.

Thirteen trials including 926 patients reported changes in tefficacy rate as the end-point outcome. Heterogeneity was low (*P* = 0.40, *I*^2^ = 4%). A fixed effects model was used for the meta-analysis. The results showed that acupuncture could significantly improve the efficacy rate of patients with LC (RR = 1.31, 95% CI [1.22, 1.40], *P* < 0.00001; Figure 3).

### 4.2. Liver Function Grading Assessment

Some liver function grading scores are widely used to assess the response to therapy, such as the Child-Pugh score, MELD score, and MELD-Na score. However, none of the 15 studies reported impact of acupuncture on liver function grading assessment.

### 4.3. Alanine Transaminase (ALT)

**Figure 4:**
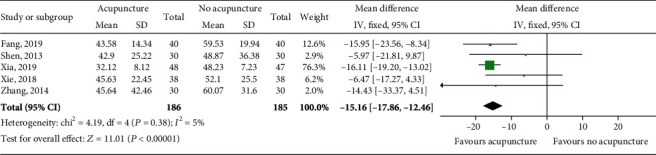
Impact of acupuncture on ALT. Both *I*^2^ and *P* represent the criteria for the heterogeneity test. ♦: pooled mean difference; —■—: mean difference and 95% CI.

Five studies had reported the ALT level in 371 patients. Heterogeneity was found to be low (*P* = 0.38, *I*^2^ = 5%), and the fixed effects model was adopted. The results showed that acupuncture combined with conventional treatment significantly reduced the level of ALT as compared with conventional therapy (MD = −15.16; 95% CI [-17.86, -12.46], *P* < 0.00001; Figure 4).

### 4.4. Aspartate Aminotransferase (AST)

**Figure 5:**
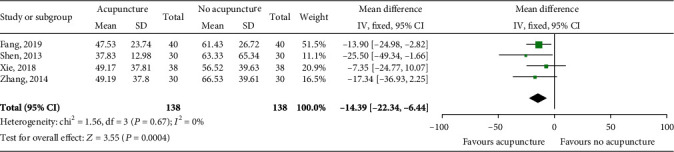
Impact of acupuncture on AST. Both *I*^2^ and *P* represent the criteria for the heterogeneity test. ♦: pooled mean difference; —■—: mean difference and 95% CI.

AST levels were reported in four studies involving 276 patients. There was no heterogeneity between studies (*P* = 0.67; *I*^2^ = 0%), and the fixed effects model was used for the meta-analysis. The results showed that when compared with the control group, acupuncture treatment resulted in a significant improvement in AST level (MD = −14.39, 95% CI [-22.34, -6.44], *P* = 0.0004; Figure 5).

### 4.5. Albumin (ALB)

**Figure 6:**
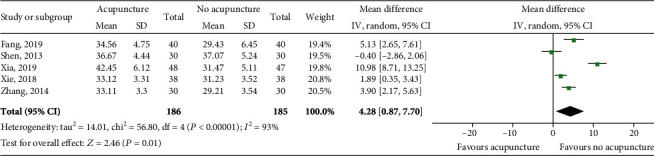
Impact of acupuncture on ALB. Both *I*^2^ and *P* represent the criteria for the heterogeneity test. ♦: pooled mean difference; —■—: mean difference and 95% CI.

Five trials including 371 patients reported data regarding this end-point. There was a high level of heterogeneity between studies (*P* < 0.00001; *I*^2^ = 93%), and the random effects model was used for meta-analysis. The results suggested that acupuncture might improve the ALB level in the patients (MD = 4.28, 95% CI [0.87, 7.70], *P* = 0.01; Figure 6).

### 4.6. Total Bilirubin (TBIL)

**Figure 7:**
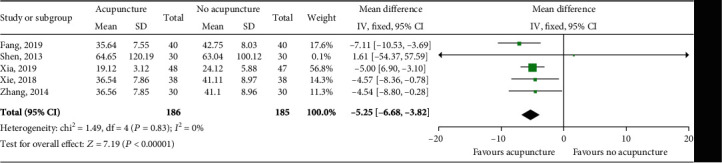
Impact of acupuncture on TBIL. Both *I*^2^ and *P* represent the criteria for the heterogeneity test. ♦: pooled mean difference; —■—: mean difference and 95% CI.

TBIL values were reported in five studies involving 371 patients. Heterogeneity was absent (*P* = 0.83, *I*^2^ = 0%). The fixed effects model was adopted. The results of meta-analysis suggested that the TBIL level of the acupuncture group was significantly lower than that of the control group, and the difference was statistically significant (MD = −5.25; 95% CI [-6.68, -3.82], *P* < 0.00001; Figure 7).

### 4.7. Bias Analysis

**Figure 8:**
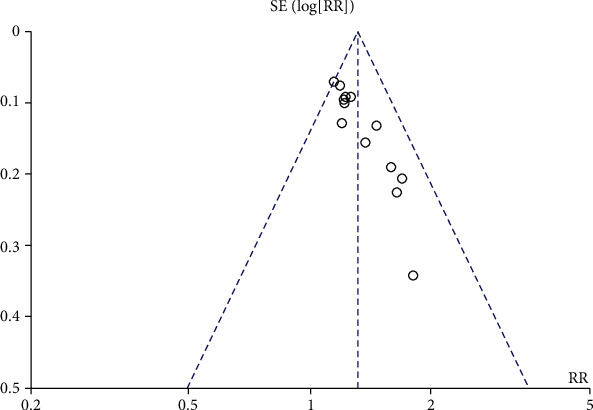
Funnel plot of acupuncture versus no acupuncture on efficacy rate.

**Figure 9:**
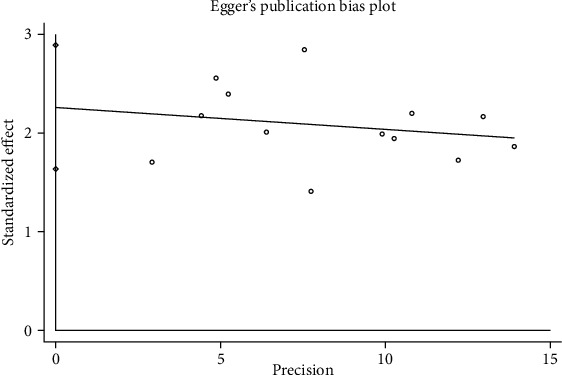
Egger's publication bias plot of the efficacy rate.

Publication bias was analyzed by a funnel plot. Possible asymmetries were found from the funnel plots (Figure 8). To further assess publication bias quantitatively, the Stata version 14.0 software for the Egger test was used. The results indicated that publication bias was not significant (*P* = 0.415; Figure 9).

## 5. Discussion

Acupuncture is often thought to be multilayered, multitargeted, and multieffective; it can benefit patients with chronic liver disease. Modern research has shown that acupuncture treatment can cause improvements at different levels, including structural, cellular, and molecular biology. The clinical effect of acupuncture is mainly reflected in improving liver function, alleviating clinical symptoms, and regulating immune function of the patients [25, 26]. The therapeutic mechanisms of acupuncture include inhibiting hepatic stellate cell activation and proliferation, reducing oxidative stress, inhibiting inflammatory response, and promoting lipid metabolism of hepatocytes [27–29]. Animal studies have confirmed that acupuncture has a positive effect on improving gastrointestinal motility and tissues of LC. Multiple animal studies reported that acupuncture promotes ECM degradation of the liver tissue, possibly related to activation of the TGF-*β*/Smad signaling pathway or inhibition of the PDGF signaling pathway. Animal studies also showed that acupuncture has beneficial effects on inflammatory responses caused by dyslipidemia through regulating contain receptors of Kupffer cells, such as scavenger receptors, complement receptors, and pattern recognition receptors. Thinning of fibrous septa, mitigation of necrosis induced by inflammatory responses, and reduction of extracellular matrix were observed in the liver after acupuncture treatment [30, 31]. More importantly, these positive effects have also been demonstrated in controlled clinical trials [32, 33]. The regulating effect of acupuncture on different cellular and molecular pathways supports its clinical application for treatment of LC.

To date, there is a lack of comprehensive systematic review and meta-analysis on the effect of acupuncture for LC treatment. The purpose of our meta-analysis was to find current evidence on the clinical application of acupuncture for this disease. It is indicated that acupuncture at specific acupoints was beneficial to patients with LC, had no hepatotoxicity and few adverse reactions, and could be used as an adjuvant treatment for LC. According to the basic theory of traditional Chinese medicine, acupuncture needles are manipulated by flicking and rotating (defined as “manual needle acupuncture”), although it is known that acupuncture manipulation, frequency, duration of needle retention, and intensity of stimulation all affect the curative effect [34]. Manual needle acupuncture was applied in all the 15 tails. Due to the need for treatment based on syndrome differentiation in clinical trials, the choice of acupoints in these trials varied greatly. In the 15 included studies, some acupoints were used more often than others, such as Zusanli (ST36), Taichong (LR3), Tanyinjiao (SP6), Ganshu (BL18), Yanglingquan (GB34), and Zhongwan (RN12).

LC is a significant challenge for physicians. Since many patients have already developed decompensation when they visit hospitals, they often have jaundiced, fatigued, insomnia, abdominal distension, and an array of other symptoms. Five of the 15 included studies reported these symptoms. Four of them reported a decrease in symptom scores after acupuncture, and one trial reported reductions in the rate of symptoms. However, because these five studies used different scoring criteria, we could not merge the results. Since these studies did not report outcome about health-related quality of life, no evidence is available about whether acupuncture improves patients' quality of life.

All the included studies involved specific efficacy criteria, such as liver function immunoglobulin indices, liver fibrosis indicators, and ascites related indexes. All of them mentioned only that acupuncture is a safe and reliable therapy, but no adverse events were reported. Therefore, the evidence on the safety of acupuncture is weak.

Despite limitations of the included studies, this meta-analysis supports the effectiveness of acupuncture for LC. It suggests that acupuncture may benefit LC patients by improving liver function and alleviating the clinical symptoms. The problems of the included studies include small sample size, limited data, and deficiencies in research methods. All of the included clinical studies lacked long-term follow-up: only one study was followed for 1 year and reported recurrence rate of ascites, while others had short follow-up periods. The quality of each research methodology was not high. Because double-blind methods and allocation concealment were not applied, it was not possible to eliminate potential placebo effect and selective bias during group assignment of participants. Since no literature with negative results was retrieved in this study, the existence of literature selection bias also could not be excluded. These factors affect the extent of the recommendation from the system of evaluation. In the future, multicenter and large sample studies with better study design should be adopted.

## 6. Conclusions

The meta-analysis suggested that acupuncture is a therapeutic option in patients with LC as a type of complementary medicine. Because the meta-analysis was based on studies with a relatively small sample, it is necessary to conduct strict, well-designed, large-scale, multicenter randomized controlled studies which further confirm the efficacy of acupuncture for LC treatment.

## Figures and Tables

**Table 1:** PICOS criteria for study selection.

Parameter	Criteria for studies
P (population)	Patients with liver cirrhosis
I (intervention)	Acupuncture
C (comparison)	No acupuncture
O (outcomes)	Efficacy rate of acupuncture therapy; impact of acupuncture on liver function grading assessment; outcomes related to liver function
S (study design)	Randomized clinical trials

**Table 2:** Characteristics of included studies.

Included study (year)	Study country	Number of subjects (*E*/*C*)	Intervening measure (*E*)	Duration (day)
Chen, 2017	China	47/40	Plus acupuncture on the basis of the control group	30
Deng, 2019a	China	25/25	Plus acupuncture on the basis of the control group	30
Deng, 2019b	China	30/30	Plus acupuncture on the basis of the control group	14
Du, 2015	China	45/45	Plus acupuncture on the basis of the control group	14
Fang, 2019	China	40/40	Plus acupuncture on the basis of the control group	30
Li, 2016	China	35/35	Plus acupuncture on the basis of the control group	10
Liu, 2018	China	40/40	Plus acupuncture on the basis of the control group	30
Qu, 2014	China	26/25	Plus acupuncture on the basis of the control group	14
Shen, 2013	China	30/30	Plus acupuncture on the basis of the control group	14
Xia, 2019	China	48/47	Plus acupuncture on the basis of the control group	28
Xiao, 2010	China	50/35	Plus acupuncture on the basis of the control group	14
Xie, 2018	China	38/38	Plus acupuncture on the basis of the control group	5
Yu, 2013a	China	40/40	Plus acupuncture on the basis of the control group	8
Yu, 2013b	China	21/21	Plus acupuncture on the basis of the control group	14
Zhang, 2014	China	30/30	Plus acupuncture on the basis of the control group	30

Note: *E*/*C* = experimental/control group.

## Data Availability

The extracted data used to support the findings of this study are included within the article. The search strategy data used to support the findings of this study are included within the supplementary information file. Other relevant data supporting this meta-analysis come from previously reported studies and cited data sets.

## Supplementary Materials

Supplementary Materials The supplementary file contains the detailed search strategy.
