# Supplementary material for: Acupuncture for the Treatment of Liver Cirrhosis: A Meta-analysis
Source: Gastroenterol Res Pract. 2020 Nov 27;2020:4054781. doi: 10.1155/2020/4054781 (PMC7737460; doi:10.1155/2020/4054781)
Supplement: Supplementary Materials — The supplementary file contains the detailed search strategy. [file 4054781.f1.docx]

Appendix 1. Search strategies

| Database | Time span | Search strategy |
| --- | --- | --- |
| PubMed | Date of database establishment to June 2020 | ((((((((("acupunct*"[Title/Abstract] OR "acupress*"[Title/Abstract]) OR "acupoint*"[Title/Abstract]) OR "electroacupunct*"[Title/Abstract]) OR "auriculotherap*"[Title/Abstract]) OR "auriculoacupunct*"[Title/Abstract]) OR "moxibust*"[Title/Abstract]) OR "meridian*"[Title/Abstract]) OR "patch*"[Title/Abstract]) AND ("Liver Cirrhosis"[MeSH Terms] OR (((("hepatic cirrhosis"[Title/Abstract] OR "cirrhosis hepatic"[Title/Abstract]) OR "cirrhosis liver"[Title/Abstract]) OR "fibrosis liver"[Title/Abstract]) OR "liver fibrosis"[Title/Abstract]))) AND (("randomized controlled trial"[Publication Type] OR "randomized"[Title/Abstract]) OR "placebo"[Title/Abstract]) |
| Cochrane Library | Date of database establishment to June 2020 | #1 MeSH descriptor: [Liver Cirrhosis] explode all trees  #2 (hepatic cirrhosis):ti,ab,kw OR (cirrhosis hepatic):ti,ab,kw OR (cirrhosis liver):ti,ab,kw OR (fibrosis liver):ti,ab,kw OR (liver fibrosis):ti,ab,kw  #3 MeSH descriptor: [Acupuncture] explode all trees  #4 MeSH descriptor: [Acupuncture Therapy] explode all trees  #5 (acupunct* or acupress* or acupoint* or electroacupunct* or auriculotherap* or auriculoacupunct* or moxibust* or meridian* or patch*):ti,ab,kw  #6 #1 or #2  #7 #3 or #4 or #5  #8 #6 and #7 |
| Embase | Date of database establishment to June 2020 | #1 'acupuncture'/exp  #2 'acupunct*':ab,ti OR 'acupress*':ab,ti OR 'acupoint*:ab,ti' OR 'electroacupunct*':ab,ti OR 'auriculotherap*':ab,ti OR 'auriculoacupunct*':ab,ti OR 'moxibust*':ab,ti OR 'meridian*':ab,ti OR 'patch*':ab,ti  #3 'liver cirrhosis'/exp  #4 'hepatic cirrhosis':ab,ti OR 'cirrhosis hepatic':ab,ti OR 'cirrhosis liver':ab,ti OR 'fibrosis liver':ab,ti OR 'liver fibrosis':ab,ti  #5 #1 or #2  #6 #3 or #4  #7 'random':ab,ti OR 'placebo':ab,ti OR 'double-blind':ab,ti  #8 #5 and #6 and #7 |
| Web of Sceice | Date of database establishment to June 2020 | TS=(acupunct* or acupress* or acupoint* or electroacupunct* or auriculotherap* or auriculoacupunct* or moxibust* or meridian* or patch*)  TS=(Liver Cirrhosis OR hepatic cirrhosis OR cirrhosis hepatic OR cirrhosis liver OR fibrosis liver OR liver fibrosis)  TS=(random* controlled trial OR random* OR placebo) |
| Wanfang Data((WANFANG)) | Date of database establishment to June 2020 | #1 'Liver Cirrhosis' OR 'hepatic cirrhosis' OR 'cirrhosis hepatic' OR 'cirrhosis liver' OR 'fibrosis liver' OR 'liver fibrosis' in subject  #2 'moxibustion' OR 'acupunture' OR 'electroacupuncture' OR 'laser acupuncture' OR 'acupressure' OR 'acupoint injection' OR 'moxibustion' OR 'patching' in subject  #3 random in subject  #4 #1 AND #2 AND #3 |
| SinoMed(CBM) | Date of database establishment to June 2020 | #1 'Liver Cirrhosis' OR 'hepatic cirrhosis' OR 'cirrhosis hepatic' OR 'cirrhosis liver' OR 'fibrosis liver' OR 'liver fibrosis' in subject  #2 'moxibustion' OR 'acupunture' OR 'electroacupuncture' OR 'laser acupuncture' OR 'acupressure' OR 'acupoint injection' OR 'moxibustion' OR 'patching' in subject  #3 random in subject  #4 #1 AND #2 AND #3 |
| China Network  Knowledge Infrastructure (CNKI) | Date of database establishment to June 2020 | #1 'Liver Cirrhosis' OR 'hepatic cirrhosis' OR 'cirrhosis hepatic' OR 'cirrhosis liver' OR 'fibrosis liver' OR 'liver fibrosis' in subject  #2 'moxibustion' OR 'acupunture' OR 'electroacupuncture' OR 'laser acupuncture' OR 'acupressure' OR 'acupoint injection' OR 'moxibustion' OR 'patching' in subject  #3 random in subject  #4 #1 AND #2 AND #3 |
| VIP medicine information system(VIP) | Date of database establishment to June 2020 | #1 'Liver Cirrhosis' OR 'hepatic cirrhosis' OR 'cirrhosis hepatic' OR 'cirrhosis liver' OR 'fibrosis liver' OR 'liver fibrosis' in title or keywords  #2 'moxibustion' OR 'acupunture' OR 'electroacupuncture' OR 'laser acupuncture' OR 'acupressure' OR 'acupoint injection' OR 'moxibustion' OR 'patching' in title or keywords  #3 random in title or keywords  #4 #1 AND #2 AND #3 |
